# Simple protoplast isolation system for gene expression and protein interaction studies in pineapple (*Ananas comosus* L.)

**DOI:** 10.1186/s13007-018-0365-9

**Published:** 2018-10-29

**Authors:** S. V. G. N. Priyadarshani, Bingyan Hu, Weimin Li, Hina Ali, Haifeng Jia, Lihua Zhao, Simon Peter Ojolo, Syed Muhammad Azam, Junjie Xiong, Moakai Yan, Zia ur Rahman, Qingsong Wu, Yuan Qin

**Affiliations:** 1 State Key Laboratory of Ecological Pest Control for Fujian and Taiwan Crops, Key Lab of Genetics, Breeding and Multiple Utilization of Crops, Ministry of Education, Fujian Provincial Key Laboratory of Haixia Applied Plant Systems Biology, Center for Genomics and Biotechnology, College of Crop Sciences, College of Resources and Environment, Fujian Agriculture and Forestry University, Fuzhou, 350002 Fujian Province China; 2 College of Life Sciences, Fujian Agriculture and Forestry University, Fuzhou, 350002 Fujian Province China; 3 South Subtropical Crops Research Institute, Chinese Academy of Tropical Agricultural Sciences, Zhanjiang, 524091 Guangdong Province China

**Keywords:** Protoplast, BAP, NAA, Transfection, Pineapple

## Abstract

**Background:**

An efficient transformation protocol is a primary requisite to study and utilize the genetic potential of any plant species. A quick transformation system is also crucial for the functional analysis of genes along with the study of proteins and their interactions in vivo. Presently, however, quick and effective transformation systems are still lacking for many plant species including pineapple. This has limited the full exploration of the genetic repository of pineapple as well as the study of its genes, protein localization and protein interactions.

**Results:**

To address the above limitations, we have developed an efficient system for protoplast isolation and subcellular localization of desired proteins using pineapple plants derived from tissue culture. A cocktail of 1.5% (W/V) Cellulase R-10 and 0.5% (W/V) Macerozyme R-10 resulted in 51% viable protoplasts with 3 h digestion. Compared to previously reported protocols, our protoplast isolation method is markedly faster (saving 4.5 h), requires only a small quantity of tissue sample (1 g of leaves) and has high yield (6.5 × 10^5^). The quality of the isolated protoplasts was verified using organelle localization in protoplasts with different organelle markers. Additionally, colocalization analysis of two pineapple Mg^2+^ transporter genes in pineapple protoplasts was consistent with the results in a tobacco transient expression system, confirming that the protoplast isolation method can be used to study subcellular localization. Further findings showed that the system is also suitable for protein–protein interaction studies.

**Conclusion:**

Based on our findings, the presently described method is an efficient and effective strategy for pineapple protoplast isolation and transformation; it is convenient and time saving and provides a greater platform for transformation studies.

## Background

Pineapple (*Ananas comosus* L.) is the second most economically important fruit crop in the world after banana, and the improvement of its qualities are aimed at increasing yield, taste and resistance to environmental changes [1]. It is a monocotyledonous triploid fruit that belongs to the family Bromeliaceae [2–4]. Pineapple exhibits crassulacean acid metabolism (CAM) and has been used as a model to study the CAM pathway [5]. The available pineapple genome sequence has also provided an opportunity to study the molecular basis of self-incompatibility [5]. Although pineapple is important as a model and crop plant, its self-incompatibility and long lifespan continue to pose challenges for breeding programs [6]. *Agrobacterium*-mediated transformation in pineapple is time consuming and technically challenging, with transformation efficiency reported to be as low as 0.12–2.69% [7]. As a result, this bottleneck in *Agrobacterium*-mediated pineapple transformation hinders crop improvement achieved through molecular breeding methods. Therefore, an easy and efficient transformation method is urgently needed to overcome these limitations and to facilitate the functional characterization of genes, the localization and interaction of proteins and transgenic studies in pineapple.

Plant protoplasts can be used as a cell-based experimental model to introduce macromolecules such as DNA, RNA and proteins using different methods such as PEG-mediated transformation, electroporation and microinjection [8, 9]. Protoplasts have been critical for studying many aspects of plant biology, including hybridization, light/chloroplast-related activities [10] and plant defense mechanisms [11]. Protoplast-based transient expression assays are very convenient as they allow rapid and high-throughput analysis of gene expression, protein subcellular localization, protein activity and protein–protein interactions [10]. Transient expression systems have been used in rice [12], *Panicum virgatum* L. [13], barley [14], grapevine [11], wheat [15], ryegrass [16] and *Arabidopsis* [17, 18] to localize gene products and to study protein activity.

Although pineapple is an important fruit crop, a homologous transient expression system has not yet been developed to facilitate the study of pineapple gene function, protein localization, protein–protein interactions, and important mechanisms. Here, we have developed a method for efficient protoplast isolation from micro-propagated pineapple plants and PEG-mediated transformation. The current method will facilitate future study in pineapple insofar as it improves upon other transformation methods, such as *Agrobacterium*-mediated transformation and biolistic bombardment. For example, the viable protoplast yield of 3.3 × 10^5^ is comparatively higher than the yield from a method previously reported by Wu et al. [18] for *Arabidopsis*, and the method requires less time than the protocol described by Zhao et al. [24] for pineapple. In the present study, we also performed protein localization analysis in isolated protoplasts and colocalized two homologous pineapple Mg^2+^ transporter genes (35S:*Aco004213.1*-GFP and 35S:*Aco004963.1*-GFP) with organelle markers. Furthermore, a BiFC assay was performed to evaluate the suitability of pineapple protoplasts for protein–protein interaction studies using previously reported JASMONATE ZIM-DOMAIN (JAZ) and MYC interacting proteins. JAZ-MYC interaction has been reported in both *Arabidopsis thaliana* [19] and *Hevea brasiliensis* [20] based on nuclear co localization by BiFC analysis and interaction by yeast two-hybrid assays. Using the proteins AtJAZ3 and AtMYC2, we found that the pineapple protoplast system is suitable for the study of protein–protein interactions.

These results suggest that protein localization and interaction studies could be performed using the current method by expressing more than one tagged construct at a time. Combined with the available genome sequencing data, this system could also facilitate research on the molecular control of pineapple plant growth. To our knowledge, this is the first successful report of a transient assay system in pineapple using protoplasts and may therefore be a useful technique for pineapple gene and protein analysis and for breeding applications.

## Materials and methods

### Plant material and culture conditions

Crown meristems and slips of three pineapple varieties (Tainong 11, MD2 and Tainong 21) were used as explants for the initiation stage of micro-propagation. The explants were washed thoroughly with commercial antibacterial liquid detergent for 30 min followed by washing with running tap water for 4 h. The explants were sterilized with 75% ethanol, followed by three washes with sterilized distilled water under aseptic conditions. The explants were divided into six sets, five of which were surface sterilized with different concentrations of Clorox (NaOCl, 10% and 15%) for different amounts of time (5, 10 and 15 min). The remaining set was surface sterilized with 0.1% mercuric chloride (HgCl_2_) for 10 min. All six sets were washed with sterilized distilled water three times. Before slicing the samples for inoculation into medium, the explants were dried on sterilized filter paper under aseptic conditions.

Bud initiation was tested with different concentrations of BAP (4 and 5 mg/l) combined with 0.2 mg/l NAA and 0.2 mg/l IAA in full strength MS [21] medium (pH = 5.8). Plantlets obtained from the subculturing process were treated with different rooting hormones (1 mg/l NAA and 1 mg/l IAA) in liquid and solid full strength MS medium for root induction. Full strength MS medium without added hormone was also tested for rooting.

Cultures were maintained under a light intensity of 3000 Lux and a day/night cycle of 8/16 h at 25 ± 2 °C in a controlled environment. All growth media used in the above experiments were sterilized by autoclaving at 121 °C for 20 min.

### Data collection and statistical analysis

During the bud induction stage, the number of successfully sterilized explants and the number of contaminated explants were counted. The number of buds initiated from sterilized explants was also counted. Three weeks after inoculation, the proliferation stage weight of the calli was measured, and the number of shoots was counted. At rooting stage, both the number and length of roots per plant were measured. A completely randomized design was used at 5% significance level, and ANOVA was performed using MINITAB 16 statistical analysis.

### Protoplast isolation

The leaves of 1-month-old plants grown on in vitro rooting medium were pooled together. The leaf tissue (1 g) was placed on a glass Petri dish and cut into 0.3–0.5 mm strips with a fresh razor blade. Using forceps, the leaf strips were immediately immersed into 20 ml enzyme solution (Table 1) that had been thermally pretreated at 55 °C for 5–10 min to inactivate nonspecific enzyme activity. For enzyme digestion, the solution was incubated for 3 h in the dark at room temperature with gentle shaking (30 rpm). The digestion mixture containing protoplasts was filtered through 100 μm nylon mesh, and the filtrate was centrifuged at 500×*g* for 10 min (ThermoScientific, Heraeus Multifuge X3R). The supernatant was carefully removed using a pipette. The pelleted protoplasts were resuspended in 2 ml WS2 solution and centrifuged at 500xg for 10 min. After repeating the WS2 wash step two times, the pelleted protoplasts were resuspended in 2 ml MMG solution. For the different experiments, the protoplast concentration was adjusted by diluting with MMG solution.

**Table 1:** Solutions for protoplast isolation and transfection

Solution name	Composition	Storage	Function
Enzyme solution	20 mM MES KOH, 1.5% (W/V) Cellulase R-10, 0.5% (W/V) Macerozyme R-10, 10 mM CaCl_2_, 20 mM KCl, 0.1% BSA (W/V) and 0.5 M Mannitol pH = 5.7	Freshly prepared	Digestion of cell wall material
WS2	154 mM NaCl, 125 mM CaCl_2_, 5 mM KCl, 2 mM MES KOH pH = 5.7	4 °C	Washing protoplasts
MMG	0.4 M Mannitol, 15 mM MgCl_2_, 4 mM MES pH 5.7	Freshly prepared	Resuspend the protoplast for counting and transfection
PEG-Ca^2+^ solution	40% (W/V) PEG 4000, 0.2 M Mannitol and 0.1 M CaCl_2_	Freshly prepared	Plasmid transformation
W1	0.5 M Mannitol, 20 mM KCL, 4 mM MES pH = 5.7	Freshly prepared	Resuspend protoplast after transfection

### Vector construction and plasmid preparation

The predicted full-length cDNA sequences of pineapple magnesium transporter genes were obtained by searching the pineapple genomic database (http://pineapple.angiosperms.org/pineapple/html/index). The complete open reading frames (ORFs) of *Aco004213.1* and *Aco004963.1* were amplified from full-length cDNA clones using PCR with gene-specific primers (Table 2). Each ORF without the stop codon was ligated into the Gateway^®^ pENTR/D-TOPO vector (Invitrogen), and the positive clones were confirmed by sequencing. The pENTR/D-TOPO vector was subsequently transferred into the destination vector pGWB505 (Invitrogen) with a GFP tag to construct the C-terminal GFP fusion proteins, followed by sequencing. The plasmid was extracted using the E.Z.N.A.^®^ Plasmid Maxi prep kit (Omega Bio-Tek, Inc., Nor-Cross, GA, USA) following the manufacturer’s recommended procedure. Plasmids containing endoplasmic reticulum (ER), Golgi body, peroxisome and plastid markers were purchased from the Department of Biochemistry, Cellular and Molecular Biology, University of Tennessee, Knoxville, USA [22].

**Table 2:** List of primers used for gene cloning and vector construction

*Aco004213.1*-F	CACCATGGCGCAGGGGTCGATGAA
*Aco004213.1*-R	AGACCCGACGAGGCCTTTGTA
*Aco004963.1*-F	CACCATGCGGCGCACGGGGCTC
*Aco004963.1*-R	CTCGAGCAAGCGCCTGCGCTT
YFPn-*AtJAZ3*-F	CACCATGGAGAGAGATTTTCTCGGG
YFPn-*AtJAZ3*-R	TTAGGTTGCAGAGCTGAGAGAAG
*AtMYC2*-YFPc-F	CACCATGACTGATTACCGGCTACAACC
*AtMYC2*-YFPc-R	ACCGATTTTTGAAATCAAACTTG

### Protoplast transfection

PEG-mediated transfection was performed following the method developed for *Arabidopsis* [17] with some modifications. Briefly, 100 μl of freshly isolated protoplasts was mixed with 4–8 μg of plasmid DNA. For the BiFC assay, we used 10–12 μg of total plasmid DNA. 110 μl volume of freshly prepared 40% PEG solution was added, and the tubes were inverted several times to mix the contents. Following 30–40 min incubation in the dark, 440 μl WS2 solution was added slowly and mixed well by gently inverting the tubes. The protoplasts were pelleted by centrifugation at 500 rpm for 4–5 min then resuspended in 200 μl W1 solution. Finally, the tubes were incubated in the dark at room temperature for 12–16 h.

### Microscopy

The total count and viability of isolated protoplasts were quantified using a hemocytometer and 0.05% fluorescein diacetate (FDA) assay under a light microscope (ZEISS Imager.A2 AX10, Japan). The transformed protoplasts were observed under a confocal laser scanning microscope (Leica TCS SP8X) for GFP, mCherry, YFP and chloroplast auto-fluorescence, with the excitation wavelengths set at 488–507 nm for GFP, 597–648 nm for mCherry and 659–749 nm for chloroplast auto-fluorescence.

## Results

### Explant selection and surface sterilization

**Fig. 1:**
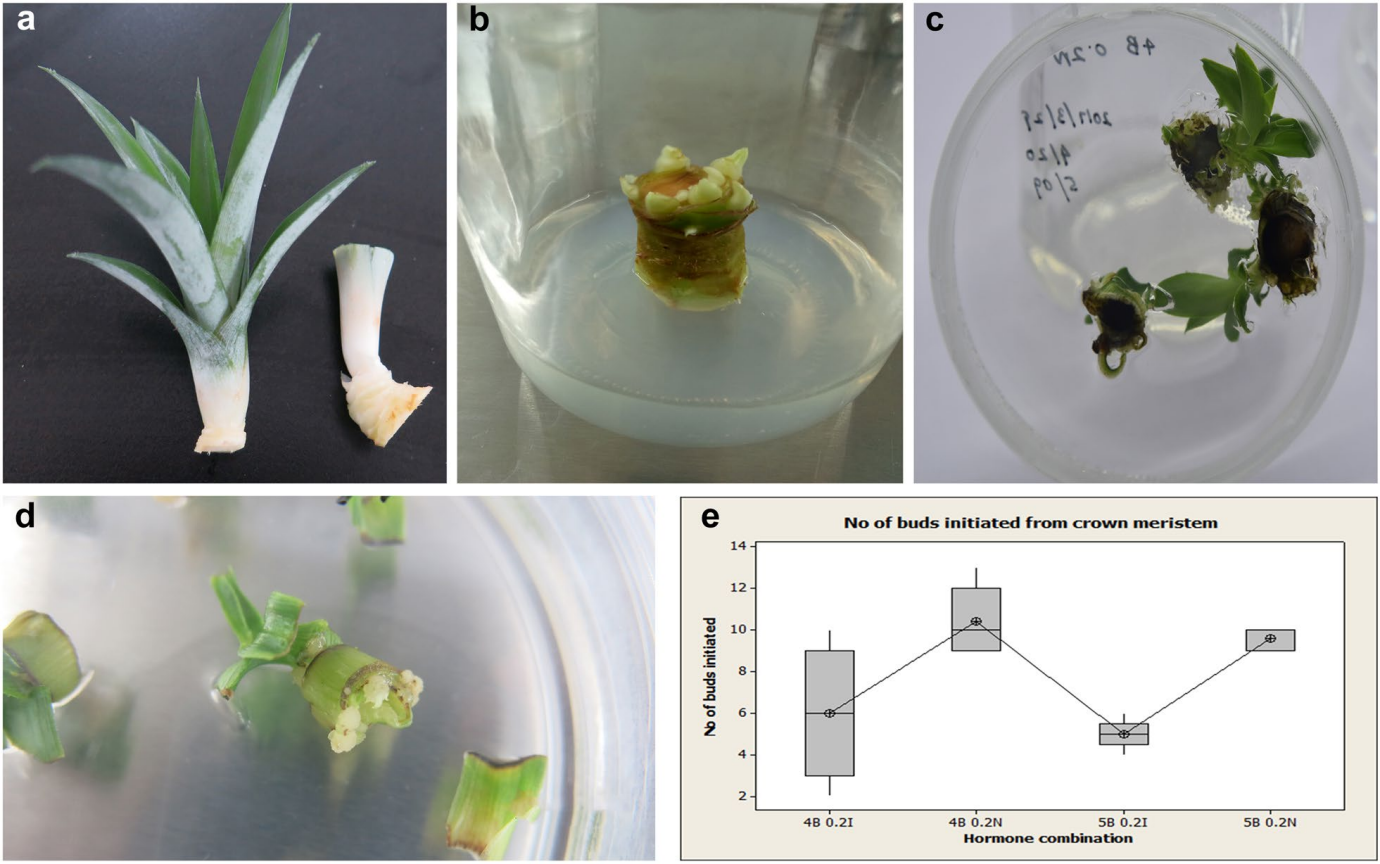
Explant selection and callus induction. **a** Crown meristem and slips prepared for surface sterilization. **b** Crown meristem with newly formed buds. **c** Stem disks with new buds. **d** Leaf bases with callus formation. **e** Box plot for the effect of different hormone combinations on bud initiation from crown meristems (95% confidence interval). 4B indicates 4 mg/l BAP; 5B indicates 5 mg/l BAP; 0.2 N indicates 0.2 mg/l NAA; 0.2I indicates 0.2 mg/l IAA

Crown meristems, stem disks and leaf bases obtained from slips were used as explants for callus induction (Fig. 1a–c). We found that 75% ethanol followed by washing with 0.1% HgCl_2_ under aseptic conditions was most suitable for pineapple explants sterilization, with approximately 75% success in callus initiation without contamination. Moreover, the age of the explant is vital for callus development. The best results (90% callus initiation) were obtained from slips less than 3 months old. All stem disks were cut to a thickness of 1–3 mm to maximize initiation.

### Callus and bud induction

Previous studies on pineapple tissue culture reported that MS medium is the most effective for organogenesis [6, 23]. We therefore used full strength MS medium in the present study during in vitro induction, subculturing and rooting. BAP is the main synthetic cytokinin used in pineapple tissue culture, and from the hormone combinations tested, we found that the best medium for the induction of callus and buds was as follows: full strength MS medium supplemented with 3% sucrose, 4 mg/l BAP and 0.2 mg/l NAA and 3 g/l phytagel. On this medium, the mean number of buds formed was 10.4 after 2 months. The hormone combination of the above medium led to a significant difference (F = 10.33 and P = 0.001) in callus and bud induction compared to the other hormone combinations (Fig. 1e). These results showed that the effect of NAA is significantly greater than the effect of IAA on bud induction.

### Hormone effect on proliferation

**Fig. 2:**
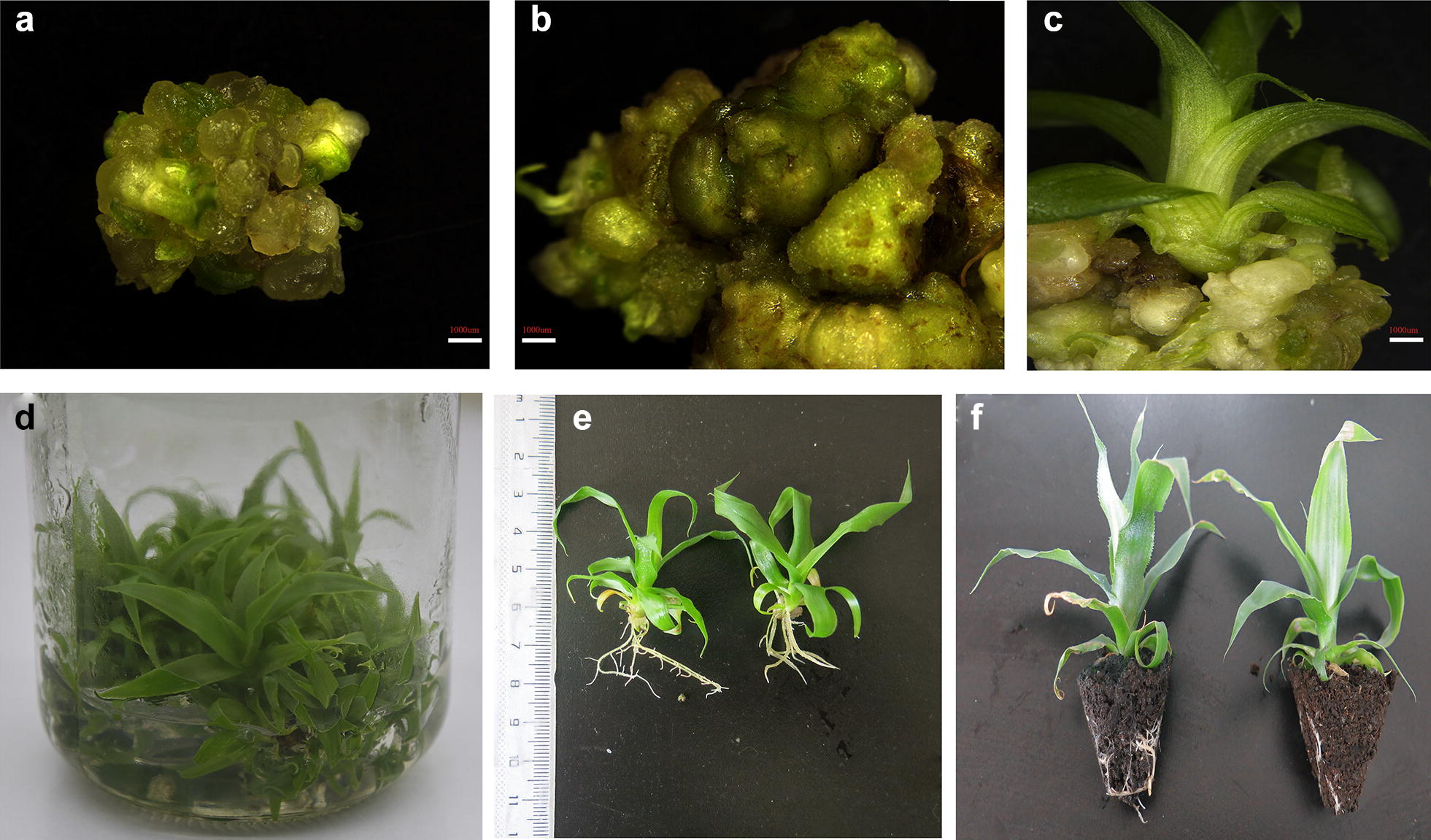
Proliferation of callus and in vitro plant growth. **a** Fragile embryonic callus. **b** Hard highly compact non-embryonic callus. **c** Small plantlets start to grow from embryonic callus. **d** Well-grown callus with small plantlets after 1 month (**e**). Plants grown in MS liquid medium for 1 month (**f**). Acclimatized in vitro grown plants. Bar in **a**–**c** = 1000 μm

We measured the weight of calli 3 weeks after inoculation to assess the effect of the different hormone combinations on callus growth. After 3 weeks, the hormone combination of 1 mg/l BAP and 0.2 mg/l NAA with full strength MS yielded the highest mean weight of 5.211 g, which was significantly different (P = 0.001 and F = 4.03) than the weights observed for the other hormone combinations (Table 3). Additionally, ANOVA indicated a significant difference (F = 6.70 and P = 0.00) in the number of shoots induced by 1 mg/l BAP and 0.2 mg/l NAA with full strength MS (9.8 shoots) compared to other hormone combinations. We observed embryonic and non-embryonic calli during the proliferation stage, with embryonic calli developing into new shoots (Fig. 2a–c).

**Table 3:** Effect of different hormone combinations on proliferation

Hormone combination	Mean weight of calli (g)	Mean no. of shoots
1 mg/l BAP 0.2 mg/l NAA	*5.221 ± 1.090* ^*A*^	*9.8 ± 1.814* ^*a*^
2 mg/l BAP 0.1 mg/l NAA	4.191 ± 1.081^AB^	6.8 ± 2.530b^c^
2 mg/l BAP 0.1 mg/l IAA	4.079 ± 1.070^B^	6.1 ± 1.912b^c^
1 mg/l BAP 0.2 mg/l IAA	3.905 ± 1.569^BC^	7.1 ± 1.663^b^
1 mg/l BAP 0.1 mg/l IAA	3.679 ± 0.950^BC^	6.8 ± 1.874^bc^
1 mg/l BAP 0.1 mg/l NAA	3.475 ± 1.417^BC^	5.1 ± 1.729^c^
2 mg/l BAP 0.2 mg/l NAA	2.965 ± 0.959^C^	5.2 ± 1.398^c^
2 mg/l BAP 0.2 mg/l IAA	2.886 ± 1.144^C^	5.1 ± 2.183^c^

Grouping information based on Fisher’s method. Means that do not share the same letter are significantly different, with a 95% confidence interval

Significance values are emphasized with italic

### Hormone-free MS medium induces in vitro pineapple rooting

In this experiment, we analyzed the effect of different media on the number of roots and root length (Fig. 2e). After 1 month, MS liquid medium supplemented with 1 mg/l of IAA gave the longest roots (average length of 2.6543 cm), while MS solid medium supplemented with 1 mg/l NAA gave the shortest roots (average length of 0.7993 cm). ANOVA revealed that root length was significantly greater on MS liquid medium supplemented with 1 mg/l IAA (P = 0.00 and F = 30.74) (Table 4).

**Table 4:** Effect of different hormone combinations on root initiation and growth

Medium type	Mean average length of roots (cm)	Mean number of roots
IAA1 mg/l liquid	*2.6543 ± 0.86* ^*A*^	3.433 ± 1.01^D^
IAA 1 mg/l solid	2.2417 ± 0.99^B^	3.867 ± 1.63^D^
*MS liquid*	*1.7817 ± 0.97* ^*B*^	*7.333 ± 1.97* ^*B*^
NAA 1 mg/l liquid	0.9647 ± 0.63^C^	5.567 ± 4.26^C^
MS solid	1.4317 ± 0.64^C^	4.233 ± 1.57^CD^
NAA 1 mg/l solid	0.7993 ± 0.24^D^	*9.23 ± 3.73* ^*A*^

Grouping information based on Fisher’s method. Means that do not share the same letter are significantly different, with a 95% confidence interval

Significance values are emphasized with italic

Considering the number of roots, MS medium supplemented with 1 mg/l NAA gave the highest number of roots compared to the other rooting media, with a significantly higher mean of 9.233 roots (P = 0.00 and F = 22.03) after 1 month (Table 4). Also taking into consideration the cost for the phytohormones, phytagel and acclimatization (Fig. 2f), we selected MS liquid medium without added hormones as the best medium for root initiation and growth, with 1.7817 ± 0.97 cm average root length and 7.333 ± 1.97 average number of roots.

### An efficient method for protoplast isolation from micro-propagated pineapple leaves

**Fig. 3:**
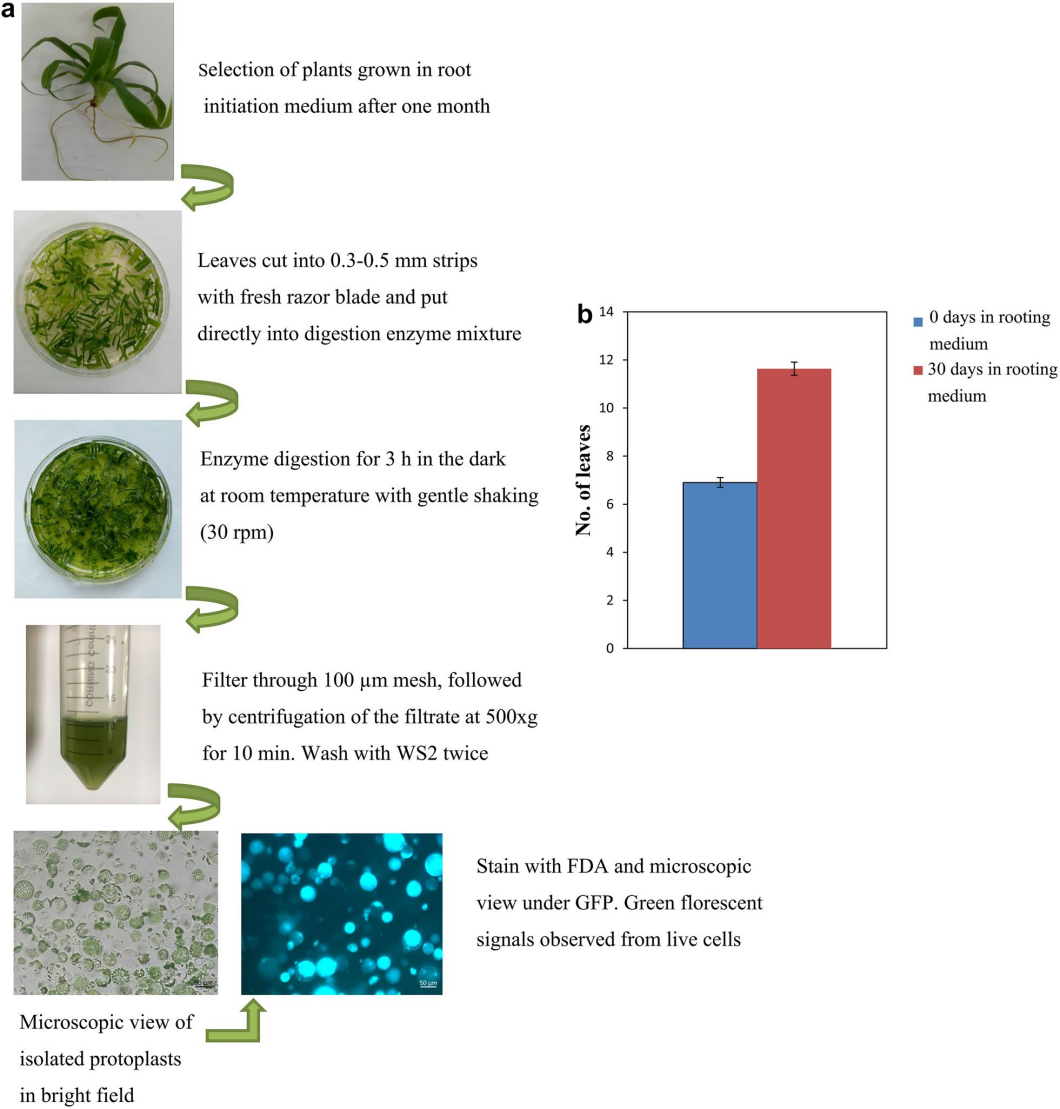
Schematic illustration of protoplast isolation. **a** Outline of protoplast isolation procedure. **b** Number of leaves at different in vitro plant growth stages

To establish an efficient protocol for pineapple protoplast isolation, we pooled together leaves from plants grown in liquid rooting medium for 1 month as the source material (Fig. 3a). Selecting leaves from the correct growth stage is critical for ensuring a high yield of protoplasts. Also, leaf yield is much greater in plants grown in rooting medium for 1 month (mean number of leaves = 11.63) than in plants directly obtained from sub culturing (mean number of leaves = 6.9) due to the significantly higher number of leaves (Fig. 3b). Green fluorescent signals observed in the protoplasts after staining with FDA indicated that the isolated protoplasts were intact and viable (Fig. 3a).

**Fig. 4:**
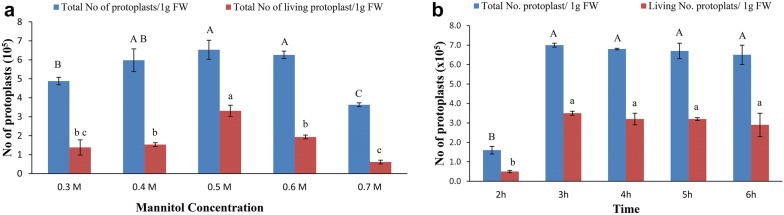
Effect of mannitol concentration and time on pineapple protoplast isolation. **a** Effect of mannitol concentration on total and living protoplast number. **b** Effect of digestion time on total and living protoplast number. Different letters represent statistically significant differences at *p *= 0.05, and bars represent standard errors

We tested the effects of different concentrations of Cellulase R-10 and Macerozyme R-10 on protoplast quantity and quality. Among the tested combinations of enzymes for digestion, the combination of 1.5% Cellulase R-10 and 0.5% Macerozyme R-10 had the highest viability (51%) (Table 5). We also examined the effect of mannitol concentration on protoplast number and viability since mannitol is important for maintaining the osmotic gradient and cell viability. The highest protoplast yield and quality were observed with 0.5 M mannitol, with a yield of 6.5 × 10^5^ protoplasts/g FW (fresh weight) (Fig. 4a).

**Table 5:** Effect of different combinations of enzymes on protoplast isolation

Enzyme combination		Total no. of protoplasts (× 10^5^)	Total no. of living protoplasts (× 10^5^)	Viability %
Macerozyme R-10%	Cellulase R-10%			
0.25	0.5	1.7 ± 0.2^d^	0.8 ± 0.05^DE^	50
0.50	0.5	1.6 ± 1.1^d^	0.6 ± 0.25^DE^	41
0.75	0.5	2.4 ± 0.6^d^	0.5 ± 0.30^E^	24
0.25	1.0	5.6 ± 0.5^ab^	2.1 ± 0.30^BC^	31
0.50	1.0	2.5 ± 0.1^d^	1.0 ± 0.33^DE^	41
0.75	1.0	2.2 ± 0.6^d^	0.7 ± 0.25^DE^	33
0.25	1.5	5.0 ± 2.8^abc^	2.5 ± 1.50^AB^	50
0.50	1.5	6.5 ± 1.4^a^	3.3 ± 0.60^A^	51
0.75	1.5	1.8 ± 1.3^c^	0.8 ± 0.57^DE^	44
0.25	2.0	4.6 ± 0.4^bc^	2.3 ± 0.26^BC^	50
0.50	2.0	4.8 ± 0.5^abc^	1.5 ± 0.17^CD^	32
0.75	2.0	3.1 ± 0.1^cd^	4.6 ± 0.16^E^	15

Grouping information based on Fisher’s method. Means that do not share the same letter are significantly different, with a 95% confidence interval

With the optimal concentrations of Cellulase R-10, Macerozyme R-10 and mannitol, we further analyzed the effects of digestion time (2–6 h). ANOVA revealed no significant differences in protoplast number or viability from 3 h digestion to 6 h digestion, but there was a slight reduction in protoplast quantity and quality (Fig. 4b). Therefore, we selected 3 h as the optimal digestion time for pineapple protoplast isolation, which contributed to the total of 4.5 h saved compared to method previously described by Zhao et al. [24]. FDA staining showed that half of the isolated protoplasts (51%) remained viable with the optimal concentrations of mannitol and digestion enzymes and the optimal digestion time (Table 5).

### Protein localization using a PEG-mediated pineapple protoplast transient expression system

**Fig. 5:**
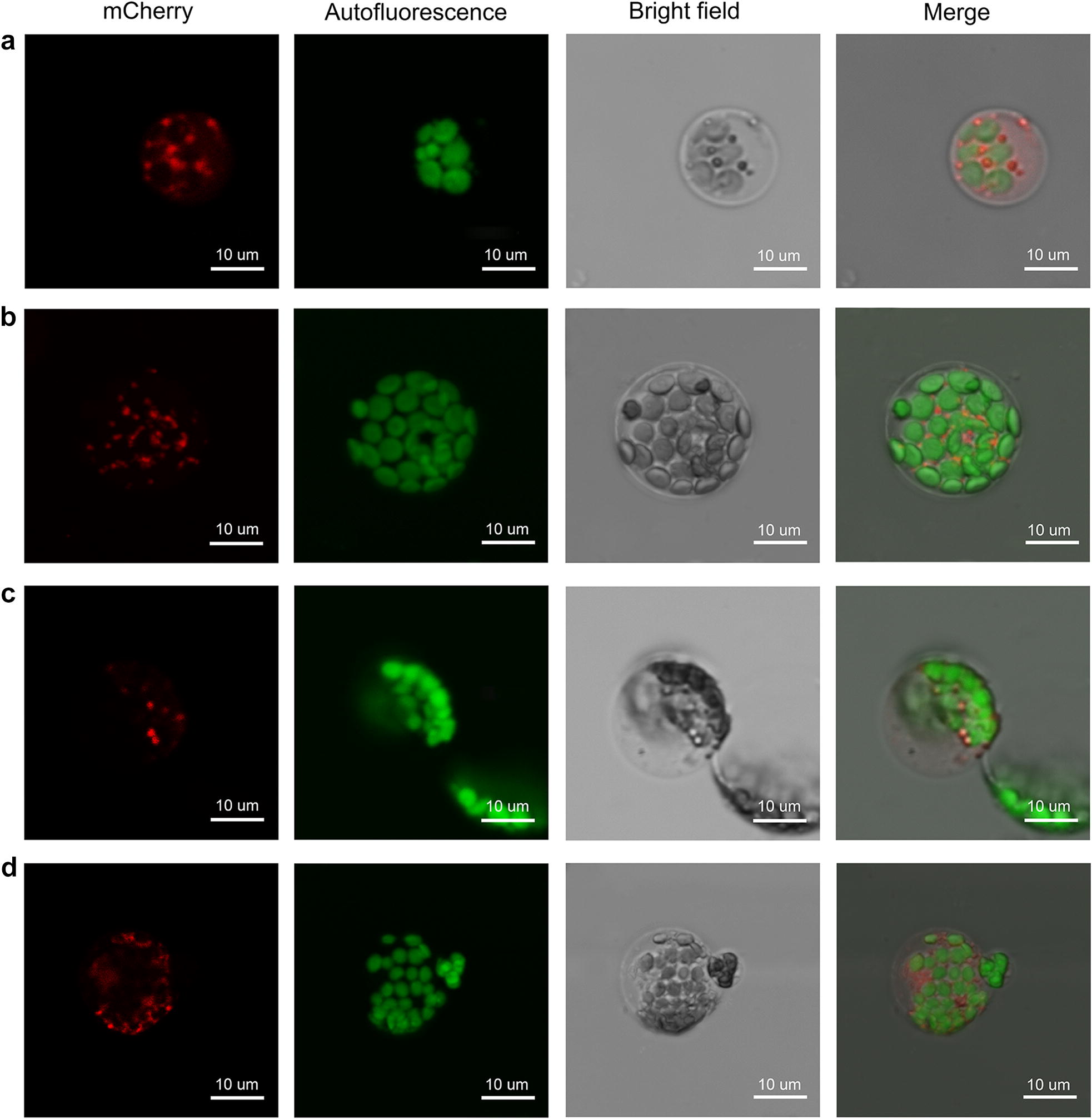
Localization of organelle markers. **a** mCherry plastid marker: pt-rk *CD3*-*999*. **b** mCherry peroxisome marker: px-rk *CD3*-*983*. **c** mCherry Golgi marker: G-rk CD3-967. **d** mCherry endoplasmic reticulum marker: ER-rk CD3-959. Bar in **a**–**d** = 10 μm

Protein fusion constructs with fluorescent proteins enable subcellular localization analysis using fluorescence microscopy [18]. The organelle markers used in this study were developed by Nelson et al. [22]. Isolated protoplasts were subjected to 40% PEG-mediated transformation with plasmids isolated using Plasmid Maxi prep kit and 12–16 h incubation. We achieved successful transformations of organelle markers tagged with mCherry, including one plastid marker (pt-rk *CD3*-*999*) (Fig. 5a), one peroxisome marker (px-rk *CD3*-*983*) (Fig. 5b), one Golgi marker (G-rk *CD3*-*967*) (Fig. 5c) and one endoplasmic reticulum marker (ER-rk *CD3*-*959*) (Fig. 5d). The observation of fluorescence in the predicted organelles demonstrated that the pineapple protoplast transient expression system is possible for the first time.

**Fig. 6:**
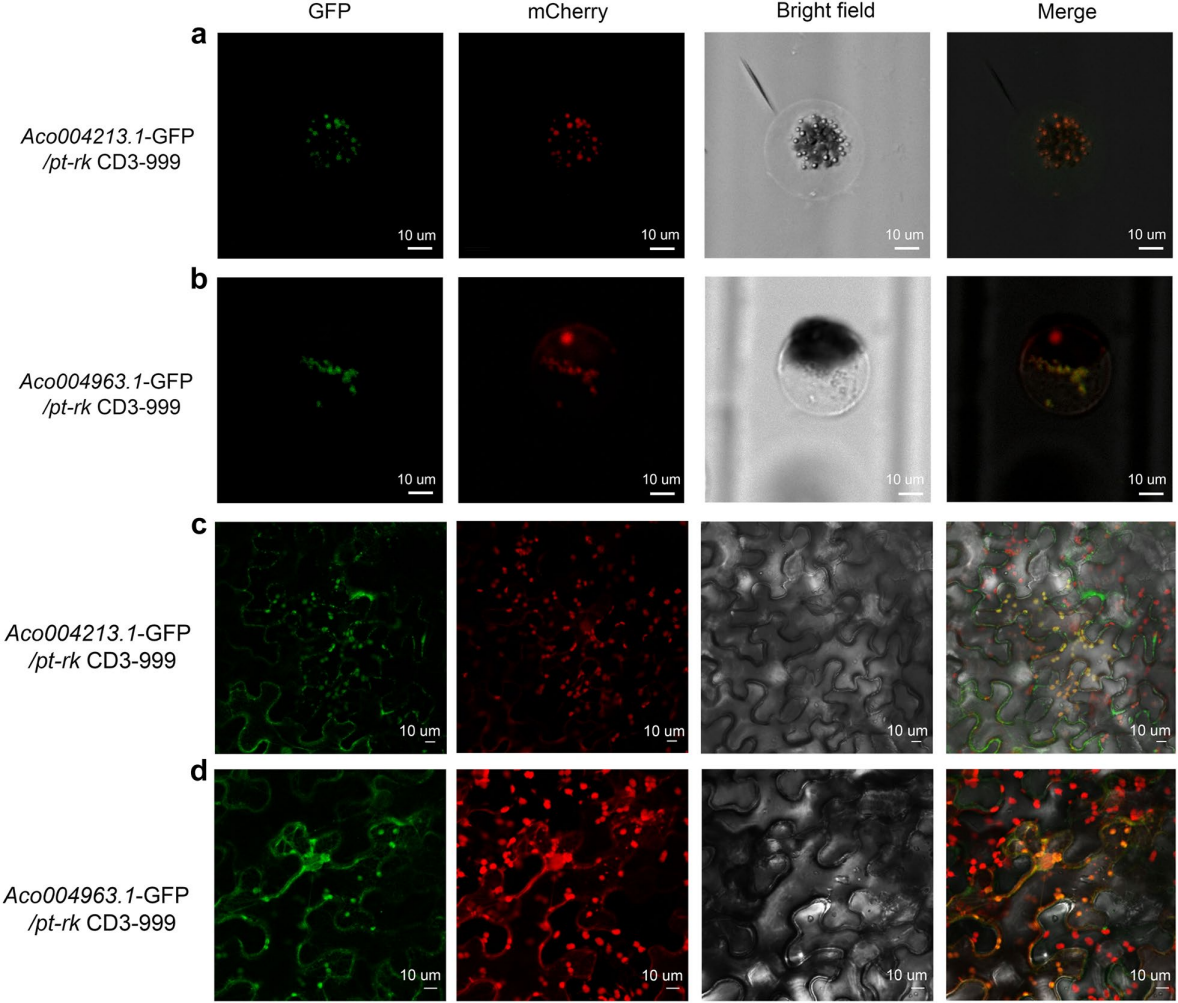
Subcellular localization of Mg^2+^ transporter genes and plastid marker in pineapple protoplasts and tobacco. **a** 35S:*Aco004213.1*-GFP and mCherry plastid marker: *pt*-*rk* CD3-999 localization in protoplast. **b** 35S:*Aco004963.1*-GFP and mCherry plastid marker: *pt*-*rk* CD3-999 localization in protoplast. **c** 35S:*Aco004213.1*-GFP and mCherry plastid marker: *pt*-*rk* CD3-999 localization in tobacco. **d** 35S:*Aco004963.1*-GFP and mCherry plastid marker: *pt*-*rk* CD3-999 localization in tobacco. Bar in **a**–**d** = 10 μm

To test the utility of the system for colocalization analysis, we used pineapple Mg^2+^ transporter genes and the organelle markers developed by Nelson et al. [22]. Both of the GFP-tagged pineapple Mg^2+^ transporter genes analyzed, *Aco004213* and *Aco004963*, colocalized with the plastid marker (Fig. 6a, b), suggesting that the gene products are present in plastids. The subcellular localization pattern of these pineapple Mg^2+^ transporter genes in the pineapple protoplast system was consistent with the localization pattern observed in tobacco (Fig. 6c, d).

### Protein–protein interaction analysis using the pineapple protoplast system

**Fig. 7:**
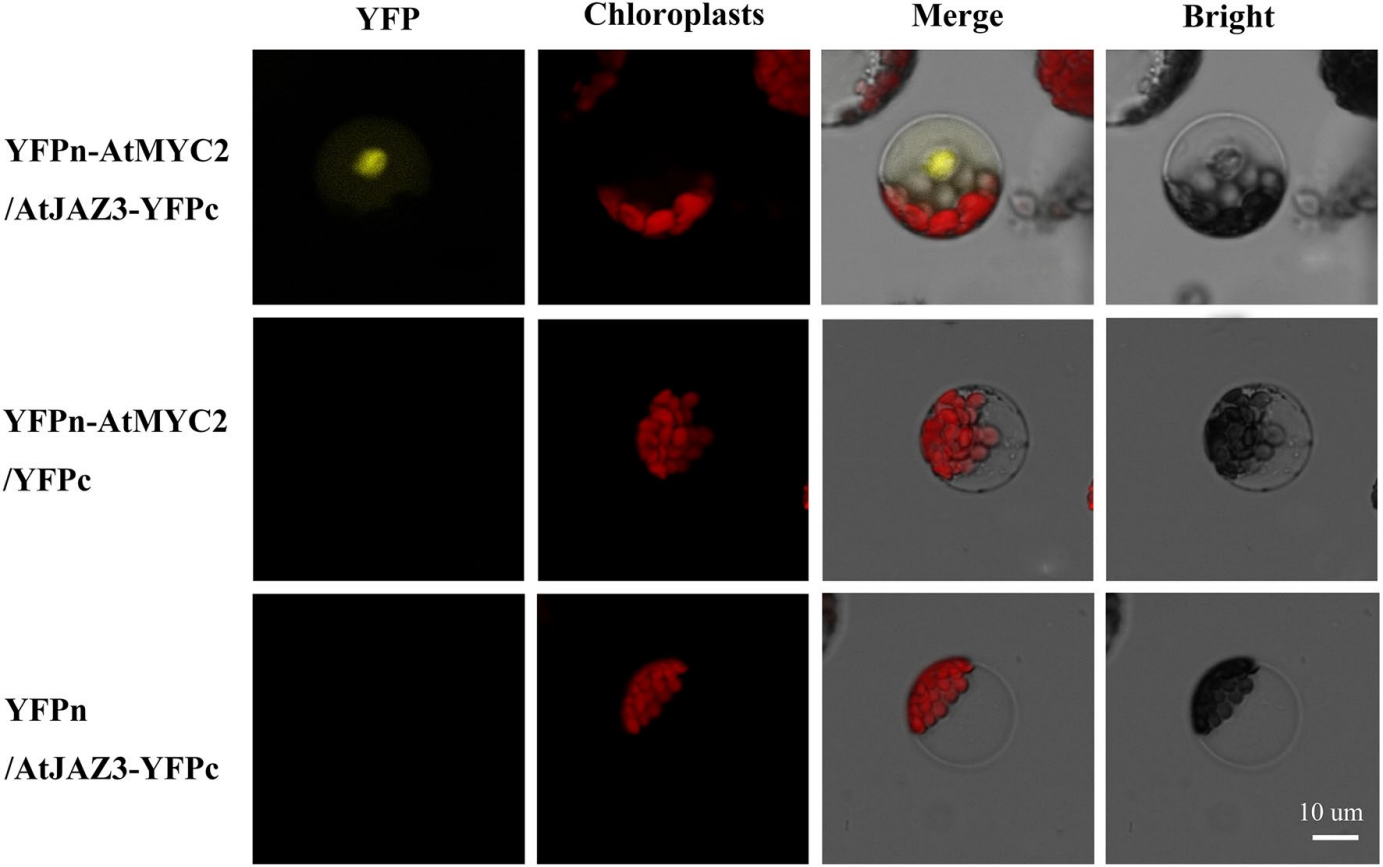
Protein-protein interaction analysis using bimolecular fluorescence complementation (BiFC) in pineapple protoplasts. Construct pairs of *YFPn*-*AtMYC2* and *AtJAZ3*-*YFPc*, *YFPn*-*AtMYC2* and empty *YFPc* vector, and empty *YFPn* vector and *AtJAZ3*-*YFPc* were transiently co-expressed in pineapple protoplasts

The ability to identify and visualize dynamic protein–protein interactions in living cells is valuable to understand cell regulatory mechanisms. We were able to successfully apply pineapple protoplast system to studying protein–protein interaction by using BIFC, one of the most powerful tools for such study. As shown in Fig. 7, co-expression of the *YFPn*-*AtMYC2* and *AtJAZ3*-*YFPc* fusion constructs resulted in a clear YFP signal in the nucleus of the protoplasts. These results are consistent with the previously reported MYC-JAZ interaction in *Arabidopsis thaliana* [19] and *Hevea brasiliensis* [20]. Empty *YFPn* vector and *AtJAZ3*-*YFPc*, *YFPn*-*AtMYC2* and *YFPc* empty vector, and empty *YFPn* and *YFPc* vectors were used as negative controls, and these combinations did not produce any fluorescent signal.

## Discussion

Protoplast transient expression systems have been developed for use in plants such as *Arabidopsis* [18] and rice [10] to study gene function, protein subcellular localization and protein–protein interactions. However, several important plants, such as pineapple, still lack an efficient protoplast transient expression system. Zhao et al. [24] previously reported a protoplast isolation method from micro-propagated pineapple leaves, but the method is limited by the amount of time and planting material required. It is also worth noting that no functional genomics studies using pineapple protoplasts have been reported thus far.

The method we describe here reduces the amount of time and source material required for pineapple protoplast isolation, and we further incorporated it into a transient expression system to facilitate protein localization and interaction studies in pineapple. Specifically, this method is 4.5 h faster than the method described by Zhao et al. [24] because it eliminates 1.5 h of pre-plasmolysis and reduces digestion time by 3 h. The amount of starting leaf material is also reduced from 3 to 1 g [24]. Therefore, this method is more efficient and effective for protoplast isolation than the previous method.

Protoplast transient expression systems rely on a high yield of isolated protoplasts with high viability from healthy plants [25]. Therefore, the proper selection of starting leaf material and maintaining the proper osmotic gradient are crucial for obtaining the high number of viable protoplasts required for subsequent steps, such as transfection and fusion. Considering that protoplasts lack cell walls, which stabilize the external and internal cellular environment by maintaining the osmotic gradient [14, 24], proper osmolarity must be maintained for protoplasts to survive [14]. We therefore determined the optimal mannitol concentration for pineapple protoplast isolation (0.5 M) in the present study. Furthermore, the use of sharp razor blades is also important because it reduces mechanical damage and crushing of cut edges.

Young plants grown in vitro are more suitable for protoplast isolation compared to mature field-grown plants due to their low fiber content and high protoplast viability. For this reason, our pineapple tissue culture system utilizes to obtain immature plantlets with less fiber. Plant tissue culture involves the generation of ‘true-to-type’ new progeny through asexual propagation [26]. Culture initiation is the most challenging critical stage due to fungal and bacterial contamination, and the use of antibiotics is discouraged due to the establishment of microbial resistance [27]. In our study, bacterial and fungal contamination during culture initiation was a major challenge, and the use of HgCl_2_ helped reduce contamination. At the rooting stage, we used hormone-free MS liquid medium to reduce the cost of the production of plants and to reduce the time required for media preparation and culturing. Therefore, the method we have developed for pineapple tissue culture is both efficient and cost effective. Previous studies have mentioned that even though pineapple micro-propagation seems easy, the multiplication rate is very slow, and it would take more than 8 years to obtain enough plantlets [28]. The method described here was able to produce more than 100,000 plants within 10 months using 10 slips for initiation.

Onion epidermal cells and tobacco leaves are commonly used for studying subcellular protein localization in plants, but different expression patterns may arise from heterologous systems [14]. Homologous systems are therefore much more highly preferred for accurate subcellular localization of proteins. Accordingly, a subcellular localization system using pineapple protoplast is a more ideal tool for studying pineapple protein localization. In the current study, mCherry-tagged markers identified four distinct organelles in the pineapple protoplasts, demonstrating that this pineapple protoplast system can be used for subcellular localization studies. We colocalized two GFP-tagged Mg^2+^ transporter genes, *Aco004213* and *Aco004963*, with mCherry-tagged plastid markers, indicating the presence of the gene products in pineapple plastids and also demonstrating that multiple marker-tagged systems can be visualized in the same protoplast. These results were consistent with the localization of the same proteins in plastids in tobacco leaves, thereby indicating that the pineapple proteins could be targeted to the correct organelles.

The nuclear co-expression of *YFPn*-*AtMYC2* and *AtJAZ3*-*YFPc* in the present study was consistent with previous reports [19, 20] and successfully demonstrated protein–protein interaction in the pineapple protoplasts. In other words, the present BiFC results using the pineapple protoplasts establish that this system is suitable for studying protein–protein interactions.

Fluorescent marker line maintenance for pineapple is not possible as in *Arabidopsis*, and pineapple transformation using *Agrobacterium* is inconsistent and time consuming and results in a low transformation efficiency [7]. Therefore, the system we have developed is fast and convenient for transgene, protein, and molecular studies with high efficacy.

## Conclusion

A highly efficient pineapple mesophyll cell protoplast isolation and transformation system was developed that can be easily used for protein subcellular localization and protein–protein interaction studies.

## Abbreviations

BAP: 6-benzylaminopurine
NAA: 1-naphthaleneacetic acid
IAA: indole-3-acetic acid
MS: Murashige and Skoog
MES: 2-(N-morpholino)ethanesulfonic acid
PEG: polyethylene glycol
ANOVA: analysis of variance
BSA: bovine serum albumin
