# Correction to: Simple protoplast isolation system for gene expression and protein interaction studies in pineapple (*Ananas comosus* L.)

**DOI:** 10.1186/s13007-018-0373-9

**Published:** 2018-12-01

**Authors:** S. V. G. N. Priyadarshani, Bingyan Hu, Weimin Li, Hina Ali, Haifeng Jia, Lihua Zhao, Simon Peter Ojolo, Syed Muhammad Azam, Junjie Xiong, Maokai Yan, Zia ur Rahman, Qingsong Wu, Yuan Qin

**Affiliations:** 1 State Key Laboratory of Ecological Pest Control for Fujian and Taiwan Crops, Key Lab of Genetics, Breeding and Multiple Utilization of Crops, Ministry of Education, Fujian Provincial Key Laboratory of Haixia Applied Plant Systems Biology, Center for Genomics and Biotechnology, College of Crop Sciences, College of Resources and Environment, Fujian Agriculture and Forestry University, Fuzhou, 350002 Fujian Province China; 2 College of Life Sciences, Fujian Agriculture and Forestry University, Fuzhou, 350002 Fujian Province China; 3 South Subtropical Crops Research Institute, Chinese Academy of Tropical Agricultural Sciences, Zhanjiang, 524091 Guangdong Province China

## Correction to: Plant Methods (2018) 14:95 10.1186/s13007-018-0365-9

In the original version of this article [1], the spelling of the tenth author name, Moakai Yan, was incorrect. The corrected name is given in this correction article. The original article has been corrected.
